# Supplementary material for: HIVprotI: an integrated web based platform for prediction and design of HIV proteins inhibitors
Source: J Cheminform. 2018 Mar 9;10:12. doi: 10.1186/s13321-018-0266-y (PMC5845081; doi:10.1186/s13321-018-0266-y)
Supplement: Supplementary file 2 — Additional file 2. Source code of HIVProtI web server. [file 13321_2018_266_MOESM2_ESM.zip › HIVprotI_Source-code/hivprotI/weka-3-6-6/doc/weka/attributeSelection/UnsupervisedSubsetEvaluator.html]

UnsupervisedSubsetEvaluator


---


|  |  |  |
| --- | --- | --- |
| **Overview** \| **Package** \| **Class** \| **Tree** \| **Deprecated** \| **Index** \| **Help** \| **Weka's home** | |  |
| **PREV CLASS**   **NEXT CLASS** | **FRAMES**    **NO FRAMES**     **All Classes** |  |
| SUMMARY: NESTED \| FIELD \| CONSTR \| METHOD | DETAIL: FIELD \| CONSTR \| METHOD |  |


---


## weka.attributeSelection Class UnsupervisedSubsetEvaluator

```
java.lang.Object
  weka.attributeSelection.ASEvaluation
      weka.attributeSelection.UnsupervisedSubsetEvaluator
```

**All Implemented Interfaces:**: java.io.Serializable, SubsetEvaluator, CapabilitiesHandler, RevisionHandler

---

``` public abstract class UnsupervisedSubsetEvaluator extends ASEvaluation implements SubsetEvaluator ```

Abstract unsupervised attribute subset evaluator.

**Version:**
:   $Revision: 1.10 $

**Author:**
:   Mark Hall (mhall@cs.waikato.ac.nz)

**See Also:**: Serialized Form

---

**Constructor Summary**

`UnsupervisedSubsetEvaluator()`


| **Method Summary** | |
| --- | --- |
| `abstract  Clusterer` | `getClusterer()`             Get the clusterer |
| `abstract  int` | `getNumClusters()`             Return the number of clusters used by the subset evaluator |
| `abstract  void` | `setClusterer(Clusterer d)`             Set the clusterer to use |

**Methods inherited from class weka.attributeSelection.ASEvaluation**

`buildEvaluator, forName, getCapabilities, getRevision, makeCopies, postProcess`

**Methods inherited from class java.lang.Object**

`equals, getClass, hashCode, notify, notifyAll, toString, wait, wait, wait`

**Methods inherited from interface weka.attributeSelection.SubsetEvaluator**

`evaluateSubset`

**Constructor Detail**

### UnsupervisedSubsetEvaluator

```
public UnsupervisedSubsetEvaluator()
```


**Method Detail**

### getNumClusters

```
public abstract int getNumClusters()
                            throws java.lang.Exception
```

:   Return the number of clusters used by the subset evaluator

    :   **Returns:**: the number of clusters used **Throws:**: `java.lang.Exception` - if an error occurs

---


### getClusterer

```
public abstract Clusterer getClusterer()
```

:   Get the clusterer

    :   **Returns:**: the clusterer

---


### setClusterer

```
public abstract void setClusterer(Clusterer d)
```

:   Set the clusterer to use

    :   **Parameters:**: `d` - the clusterer to use


---


|  |  |  |
| --- | --- | --- |
| **Overview** \| **Package** \| **Class** \| **Tree** \| **Deprecated** \| **Index** \| **Help** \| **Weka's home** | |  |
| **PREV CLASS**   **NEXT CLASS** | **FRAMES**    **NO FRAMES**     **All Classes** |  |
| SUMMARY: NESTED \| FIELD \| CONSTR \| METHOD | DETAIL: FIELD \| CONSTR \| METHOD |  |


---
